# Dementia caregiving in India: New evidence from a National representative sample

**DOI:** 10.1002/alz.70266

**Published:** 2025-05-15

**Authors:** Marco Angrisani, Nicholas S. Reed, Joyita Banerjee, Jinkook Lee

**Affiliations:** ^1^ Center for Economic and Social Research University of Southern California Los Angeles California USA; ^2^ Department of Economics University of Southern California Los Angeles California USA; ^3^ Optimal Aging Institute NYU Grossman School of Medicine New York New York USA; ^4^ Department of Otolaryngology NYU Grossman School of Medicine New York New York USA

**Keywords:** Alzheimer's disease and related dementia, cognitive impairment, dementia caregiving, India

## Abstract

**INTRODUCTION:**

As India's population ages, the prevalence of dementia is increasing rapidly, inducing higher need for informal caregivers for a complex population. However, the effects of individuals' cognitive impairment on their caregivers' well‐being in India have not been well studied.

**METHODS:**

We analyzed data from 4196 informants of participants in the Longitudinal Aging Study in India‐Diagnostic Assessment of Dementia (LASI‐DAD). Informants' caregiving roles and well‐being, including stress, mental health, positive affect, and spirituality, were assessed alongside the cognitive function and dementia status of LASI‐DAD respondents.

**RESULTS:**

Informants of individuals with cognitive impairment experienced significantly higher stress, poorer mental health, and lower levels of positive affect and spirituality. Greater caregiving responsibility intensified the negative impact on well‐being.

**DISCUSSION:**

These results demonstrate the significant emotional and psychological strain on dementia caregivers in India, underscoring the need for targeted interventions and support structures.

**Highlights:**

- We used a dual data collection approach in LASI‐DAD to create a first‐of‐its‐kind linked dataset on care recipients' and caregivers' outcomes, representative of Indian older adults.
- Using this dataset, we tested whether cognitive impairment in care recipients increased informants' stress and lowers well‐being, with greater caregiving responsibility amplifying these effects.
- Informants of cognitively impaired individuals reported significantly higher stress, poorer mental health, and lower positive affect and spirituality, with stronger effects for those with greater caregiving roles.
- Non‐primary caregivers also experienced well‐being declines when assisting a cognitively impaired older adult, likely driven by family disruptions and added household responsibilities for co‐residents and logistical challenges and reduced control for those living separately.
- Our findings highlight the widespread emotional and psychological strain of dementia caregiving in India, emphasizing the need for targeted support and interventions.

## 1 INTRODUCTION

India is experiencing a rapid demographic shift such that the percentage of adults over 60 years will double from 10% to 20% of the total population in the next 30 years,^1^ which will contribute to an increase in the prevalence of numerous chronic health conditions, including dementia.^2^ Informal caregivers, consisting of familial and friend networks, are an important resource for assisting older adults with chronic conditions and disabilities with daily and health‐related activities. Although informal caregivers play a crucial role in complementing and/or supplementing the formal healthcare workforce for older adults, there has been relatively little research on the nature of their activities, the burdens and benefits they experience, and their overall health, particularly in relation to the diverse health needs of care recipients.

The majority of the epidemiologic and surveillance evidence on informal caregiving is derived from the United States and Western Europe, which are culturally and demographically distinct from India. Research from the World Health Organization suggests India has a higher reported prevalence of informal caregivers than other low‐ and middle‐income countries (LMICs).^3^ In India, caregiving for adults with dementia is associated with reduced work hours and poorer health.^4^ Moreover, among Indian informal caregivers, more caregiving hours are associated with a higher risk of depressive symptoms.^5^ However, the current evidence on caregiving in India is mostly based on smaller, geographically restricted samples, prone to selection bias. These studies lack longitudinal insights and do not employ multilevel data collection that tracks both the caregiver and care recipient, limiting a comprehensive understanding of the caregiving experience.

To that extent, the second wave of the Harmonized Diagnostic Assessment of Dementia Longitudinal Aging Study of India (LASI‐DAD),^6^ a nationally representative longitudinal study of aging in India surveying individuals aged 60 and older, added a specific module to its existing informant questionnaire to prospectively collect data on informants' experiences and well‐being as they relate to their caregiving responsibilities. Specifically, the added questions elicited the role of informants as caregivers of LASI‐DAD respondents, their perceived level of stress, mental health status, positive affect, and spirituality. Importantly, for each of its respondents, LASI‐DAD provides rich background information on demographics, health, and socioeconomic factors, alongside unprecedented high‐quality data on cognitive performance, as assessed by a comprehensive and standardized cognitive test battery, and a clinical consensus rating of dementia. The combination of respondent‐ and informant‐level information constitutes a first‐in‐kind linked dataset of care recipients' and caregivers' outcomes.

Exploiting such a unique and population‐representative dataset, covering the entire Indian territory, we conducted an observational cross‐sectional analysis to characterize informal caregiving in India, its consequences for individual stress and well‐being, and the extent to which these outcomes vary by the care recipient's level of cognitive impairment.

We posed two hypotheses, which, given the data at our disposal, can be reliably tested for the first time in India: (1) cognitive impairment of care recipients is associated with higher levels of stress and lower levels of well‐being among informants, who are often co‐residing family members; (2) the adverse effect of respondents' cognitive impairment on informants' outcomes is more pronounced the more caregiving responsibilities the informants have.

## 2 METHODS

### 2.1 Sample and data

The LASI is an ongoing cohort study of over 73,000 Indian adults aged 45 and older, representative of the nation and each state.^7^ Drawing its sample from the LASI, LASI‐DAD constitutes the first nationally representative study specifically developed to better understand the determinants of late‐life cognition, cognitive aging, and dementia in India.^6^ Two waves of LASI‐DAD data collection have been completed. Wave 1 included 4096 respondents; Wave 2 included 4635 respondents, of which 2565 participated in both waves, yielding a 63% follow‐up rate.

LASI‐DAD comprehensively assessed respondents' cognitive function across different domains via a wide range of cognitive tests. An informant report was also obtained, typically completed by a respondent's family member. In addition, LASI‐DAD provided a Clinical Dementia Rating (CDR) for its participants.^8^ A CDR score was available for 2528 respondents in Wave 1 and 4524 respondents in Wave 2. To assess physiological markers of aging, anthropometric and physical measures were obtained in both waves of LASI‐DAD. Specifically, a comprehensive geriatric assessment battery was administered to elicit limitations in activities of daily living (ADLs) and instrumental activities of daily living (IADLs), as well as health history and doctor‐diagnosed conditions.

**RESEARCH IN CONTEXT**

1. **Systematic review**: We reviewed the existing literature on caregiving for individuals with dementia, particularly in LMICs. While prior studies from high‐income countries (HICs) highlight the psychological and emotional toll of caregiving, the evidence from India has been limited to small, geographically restricted samples lacking in longitudinal data. Moreover, no study in India or other LMICs collected prospective data about both care recipients (typically the survey respondents) and caregivers. Our study aims to fill this gap by providing population‐level insights into caregiving in India using data from the LASI‐DAD, a nationally representative study of aging and dementia. Specifically, we added a caregiver module to the LASI‐DAD informant questionnaire to elicit informants' caregiving roles and well‐being, including stress, mental health, positive affect, and spirituality, and linked this information to LASI‐DAD respondents' cognitive function and dementia status. We searched PubMed until September 1, 2024, for studies on dementia caregiving in India and other LMICs using keywords “caregivers,” “caregiving,” “dementia,” “cognitive impairment,” “India,” and “low income,” without language or publication date restrictions.
2. **Interpretation**: Our study provides the first analysis of the impact of cognitive impairment on caregiver well‐being in India based on a large, representative sample of the Indian population. We found that cognitive decline in care recipients significantly affects caregivers' stress, mental health, positive affect, and spirituality. The negative effects were most pronounced for primary caregivers, but non‐primary caregivers also experienced substantial declines in well‐being. These findings align with international studies, while also revealing unique cultural challenges faced by Indian caregivers, such as the lack of formal support systems.
3. **Future directions**: The study underscores the need for culturally sensitive dementia care policies and support structures for caregivers in India. Future research should focus on collecting longitudinal data on both care recipients and caregivers and explore the long‐term effects of caregiving on well‐being. Moreover, comparative research across LMICs could enhance our understanding of caregiving challenges in diverse cultural settings and inform global health policy.

The informant questionnaire was modified in Wave 2 to add a caregiver module. These additional questions elicited the informant's role/responsibility in assisting the respondent with their daily activities and several well‐being measures, such as stress, mental health, positive affect, and spirituality.

For our analysis, we considered Wave 2 informants who completed the caregiving module (*N* = 4474). Among these, we excluded those for whom respondent‐level key variables (CDR and cognitive test scores, ADLs) were missing. The resulting analytic sample included *N* = 4196 informants with linked respondents' information. We did not find evidence of systematic demographic differences between LASI‐DAD respondents represented in our sample and those excluded because either their informants did not complete the caregiving module or they had missing CDR/cognitive test scores or ADL information.

### 2.2 Caregivers' outcomes

The caregiver section of the informant questionnaire begins with the following question: “Are you primarily responsible for assisting (the LASI‐DAD respondent) with their daily activities, or is there someone else in the home who takes on the primary caregiving role?” The responses include the following options: (i) “I am the primary caregiver;” (ii) “I share responsibility with other family members;” (iii) “Someone else in the home is the primary caregiver;” and (iv) “The respondent primarily cares for themselves.” These options outline varying levels of involvement in caregiving, ranging from those who are the primary caregivers to those who report that the respondent is largely self‐sufficient. In our analysis, we will consider the latter group as “non‐caregivers.” Throughout the manuscript, we will use the terms “caregiver” and “caretaker” interchangeably.

Informants' stress was measured using four items from the Perceived Stress Scale.^9^ The scores across these items were summed to create a composite stress index, with higher values indicating higher stress levels. Mental health was assessed through the Center for Epidemiological Studies Depression Scale (CESD).^10^ The scores of five items were combined to form a composite mental health index, with higher values indicating poor mental health. Additionally, the module included two questions on positive emotions, asking how often in the past month the informant felt cheerful and calm/peaceful, with response options being 0 = never, 1 = occasionally, 2 = once a week, 3 = some days in a week, 4 = every day. These responses were summed to create a positive affect index, with higher values indicating more positive affect. The questionnaire also included three items assessing how frequently informants experienced feelings of inner peace, spirituality, and thankfulness. Responses were recorded on a 5‐point scale (0 = never, 1 = occasionally, 2 = once a week, 3 = some days a week, 4 = every day) and were summed to construct a spirituality index. Higher values indicate higher values of spirituality.

We created indicators taking a value of 1 if the composite indices exceed the sample median and 0 otherwise. These indicators reflect high (above median) levels of stress, poor mental health, positive affect, and spirituality, serving as the primary outcome variables in our analysis. To assess the robustness of our findings, we also conducted the analysis using the composite indices for stress, poor mental health, positive affect, and spirituality as continuous dependent variables, standardizing them to have a mean of 0 and a standard deviation of 1 within the sample.

### 2.3 Measures of respondents' cognition and dementia

LASI‐DAD administered a detailed neuropsychological battery based on the Harmonized Cognitive Assessment Protocol, which was designed within the Health and Retirement Study to measure key cognitive domains, including attention, memory, executive function, language, and visuospatial function.^11^ The protocol, adapted to accommodate relatively low levels of literacy and numeracy in India, featured an in‐person interview and one with an informant nominated by the respondent.^12^

LASI‐DAD developed a web‐based platform for clinicians to rate participants on the CDR scale.^8^ The validity of this online clinical consensus diagnosis of dementia in India was demonstrated previously.^13^ With access to demographics, cognitive test scores, information about ADLs and IADLs, and the informant report about the respondent's cognitive functioning,^14^, ^15^ CDR‐trained Indian clinicians assigned LASI‐DAD participants a score based on the CDR scoring algorithm.^8^ Specifically, the score took values of 0 for no dementia, 0.5 for questionable dementia, 1 for mild cognitive impairment, 2 for moderate cognitive impairment, and 3 for severe cognitive impairment.

### 2.4 Statistical analysis

Our empirical analysis was conducted in two stages. First, we examined whether and to what extent the respondent's potential cognitive impairment and dementia influenced informants' outcomes, specifically stress, poor mental health, positive affect, and spirituality. We tested the hypothesis that informants of respondents with cognitive impairment would exhibit lower levels of well‐being, characterized by higher perceived stress, poorer mental health, and lower levels of positive affect and spirituality. To this end, we estimated logit regression to assess how the probability of reporting high levels (above median) of stress, poor mental health, positive affect, and spirituality varies based on the respondents' CDR classifications. Specifically, we included indicators for CDR levels of questionable and mild or moderate cognitive impairment, omitting the group with a CDR of zero to estimate outcomes relative to informants of dementia‐free respondents (no LASI‐DAD respondent in Wave 2 had a CDR score of 3 indicating severe cognitive impairment).

In the second stage of the analysis, we investigated whether the impact of the respondent's cognitive impairment on the informant's well‐being differed depending on the informant's caregiving role. Our hypothesis was that greater involvement as a caregiver would be associated with a more significant negative effect of the respondent's cognitive impairment on the informant's well‐being. For this purpose, we used the same logit models as before but introduced interactions between the informant's caregiving role indicators and an indicator for the respondent's probable dementia, which was set to 1 when the respondent's CDR was greater than zero. Given the relatively small number of respondents classified as mildly or moderately impaired, we grouped respondents into two categories – those without impairment (CDR = 0) and those with impairment (CDR > 0) – to increase statistical power. As noted earlier, we also estimated linear regression models using the composite indices of stress, poor mental health, positive affect, and spirituality as continuous dependent variables. The results from these linear models are provided in the Supplemental Material.

In all regression models, we controlled for informants' sex, age, education, relationship to the respondent (son, daughter, daughter‐in‐law, other, with spouse/partner as the reference group), frequency of contact with the respondent (daily contact, several times a week or less frequent contact, with co‐residence as the reference group), and level of responsibility as a caregiver (primary caregiver, shared caregiver, non‐primary caregiver, with non‐caregivers as the reference group). We also adjusted for respondent sex, age, urban or rural residence, and number of ADLs. After excluding observations with missing values for any of the informants' outcomes or key independent variables, the final analytic sample consisted of 4196 informants. All analyses were performed using Stata 18.^16^

### 2.5 Role of funding source

The funder of the study had no role in study design, data collection, data analysis, data interpretation, or writing of the report. All authors had final responsibility for the decision to submit for publication.

## 3 RESULTS

### 3.1 Sample characteristics

The characteristics of LASI‐DAD informants are summarized in Table 1. Notably, the proportion of females was significantly higher than that of males (61% vs 39%). The sample included a broad age range, with the largest groups being middle‐aged individuals, specifically those aged 30 to 39 and 40 to 49. Approximately one‐quarter of the sample had no formal education, while 46% had completed secondary education or higher. Reflecting typical familial structures in India, most informants were the respondents' spouses/partners (24%), sons (23%), or daughters‐in‐law (28%). Most informants (83%) co‐resided with the LASI‐DAD respondent, while 14% had daily contact, and only 4% interacted with the respondent several times a week or less frequently. Regarding caregiving roles, 37% of informants identified as the primary caregivers, assuming full responsibility for the respondent's daily needs. Around one‐fifth shared caregiving responsibilities, while only 6% reported lesser involvement in assisting the respondent (non‐primary caretakers). The remaining third of the sample indicated that the respondent was largely self‐sufficient. As noted earlier, these informants will be classified as “non‐caregivers” in our analysis.

**TABLE 1:** Informants' characteristics.

Sex: perc (sd)	
Male	39.94% (48.76)
Female	61.06% (48.76)
Age: perc (sd)	
18–29	18.04% (38.46)
30–39	24.26% (42.87)
40–49	22.04% (41.46)
50–59	11.89% (32.37)
60–69	13.37% (34.04)
70+	10.40% (30.52)
Education: perc (sd)	
No schooling	23.96% (42.68)
Less than secondary	30.36% (45.99)
Secondary or higher	45.68% (49.82)
Stress Index (0–16)	
mean (sd)	6.13 (2.90)
Mental Health Index (0–20)	
mean (sd)	10.14 (3.08)
Relationship with R: perc (sd)	
Spouse/partner	24.14% (42.80)
Son	22.90% (42.03)
Daughter	8.87% (28.43)
Daughter‐in‐law	28.53% (45.16)
Other	15.56% (36.25)
Frequency of contacts with R: perc (sd)	
Co‐residence	82.62% (37.89)
Daily	13.62% (34.29)
Several times a week or less	3.76% (19.04)
Role as R's caretaker: perc (sd)	
Primary caretaker	36.76% (48.22)
Shared caretaker	24.16% (42.81)
Non‐primary caretaker	6.43% (24.54)
Non‐caretaker	32.65% (46.90)
Positive Affect Index (0‐8)	
mean (sd)	4.83 (1.89)
Spirituality Index (0‐12)	
mean (sd)	7.64 (3.16)

As far as informants' well‐being measures are concerned, the stress index ranged from 0 to 16, with a mean of 6.13 and a median of 6. Although the theoretical CESD score was between 0 and 20, the lowest observed value in the sample was 5, with a mean of 10.13 and a median of 10. The positive affect index spanned 0 to 8, averaging 8.83 with a median of 5. Lastly, the spirituality index, ranging from 0 to 12, had a mean of 7.64 and a median of 8.

We observed substantial variation in respondents' cognitive performance and prevalence of ADLs across informants with different caregiving roles. As shown in Table SM1 and Figure S1 in the Supplemental Material, respondents identified by their informants as “primarily caring for themselves” generally performed better in cognitive tests. However, there was significant cross‐sectional variation within this group. Notably, extremely low test scores – indicative of probable cognitive impairment – were not uncommon. In fact, according to the CDR, around 40% of respondents in this group were classified as having questionable, mild, or moderate cognitive impairment. Cognitive test performance among the three caregiver groups was similar overall. The proportion of respondents with probable dementia (CDR > 0) was 52% among primary caregivers, 56% among shared caregivers, and 54% among non‐primary caregivers, with the only statistically significant difference being between primary and shared caregivers. The variation in CDR score observed across groups of informants is crucial in understanding how respondents' cognitive impairment impacts informants' well‐being, depending on their level of responsibility as caregivers.

As shown in Table SM1, the average number of respondents' ADLs was only slightly higher among primary and shared caregivers (1.13 and 1.16, respectively) compared to non‐primary caregivers and non‐caregivers (1.07 and 1.00, respectively). Among non‐caregivers, there was a lower prevalence of respondents' difficulties with bathing and eating (Figure SM2 in the Supplemental Material). However, the prevalence of respondents' difficulties with other ADLs, such as dressing, getting in and out of bed, and using the toilet, was similar to that observed within the caregiver groups. It is likely that informants who reported the respondent to be largely independent did so due to a lack of awareness of possible limitations and chronic conditions, which aligns with their minimal involvement as caregivers.

### 3.2 The association between respondents' cognitive impairment and informants' well‐being

We evaluated informants' well‐being using four distinct outcomes: perceived stress, poor mental health, positive affect, and spirituality. For the main analysis, we created indicators for high levels of these outcomes – defined as composite index values above the sample median – and examined how the probability of experiencing elevated levels of each outcome relates to the respondent's cognitive functioning. In supplemental analyses (detailed in the Supplemental Material), we explored this relationship using the composite indices as continuous dependent variables.

**FIGURE 1:**
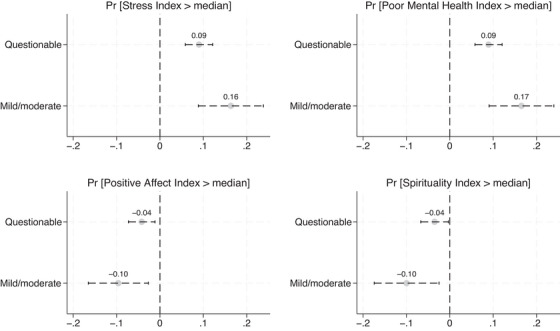
*N* = 4196. The figure reports Logit marginal effects (and corresponding 95% confidence intervals) of indicators for CDR = questionable impairment, CDR = mild or moderate impairment. The omitted indicator is the one corresponding to CDR = no impairment. All Logit regressions include controls for informants' sex, age, education, relationship with respondent, frequency of contact with the respondent, and role as respondent's caretaker. The regressions also include controls for respondents' sex, age, rural residence, and number of ADLs. The full set of estimated Logit marginal effects is in Table SM2 in the Supplemental Material. ADLs, activities of daily living; CDR, clinical dementia rating.

The richness of the LASI‐DAD data allowed us to examine how a medical diagnosis of cognitive impairment influenced informants' outcomes. As described earlier, CDR‐trained Indian clinicians assigned LASI‐DAD participants a CDR score: 0 for no dementia, 0.5 for questionable dementia, 1 for mild cognitive impairment, 2 for moderate cognitive impairment, and 3 for severe cognitive impairment. Overall, 51% of respondents had no cognitive impairment; 44.5% had questionable, 4% had mild, and 0.5% had moderate impairment. No respondent was classified as having severe cognitive impairment (CDR score of 3). We created indicators for questionable and mild or moderate cognitive impairment and regressed informants' outcomes on these indicators, controlling for informant and respondent demographics and characteristics. Figure 1 presents the estimated logit marginal effects of the questionable and mild/moderate dementia indicators separately for the probability that the informant exhibits high levels of stress, poor mental health, positive affect, and spirituality (the full set of estimated logit marginal effects is in Table SM2 in the Supplemental Material). With informants of respondents who had a CDR score of zero as the reference group, these marginal effects reflect the differences in well‐being between informants of respondents with cognitive impairment and those without it. Our hypothesis – that informants of respondents with lower cognitive function experience lower levels of well‐being – is supported when these differences are large in value and statistically significant.

Compared to informants of dementia‐free respondents, those of respondents with questionable and mild or moderate dementia experienced increases in the probability of reporting high levels of stress of 9 (*p* < .01) and 16 (*p* < .01) percentage points (pp), respectively. The difference between these two coefficients is sizeable (7pp) and statistically significant (*p* < .05), indicating an apparent gradient in informants' stress corresponding to the severity of the respondents' cognitive impairment.

A similar pattern emerged for the likelihood that the informants exhibit poorer mental health. Compared to informants of dementia‐free respondents, those of respondents with questionable and mild or moderate dementia saw an increase in the likelihood that their CESD score would be above the sample median of 9pp (*p* < .01) and 16pp (*p* < .01), respectively, highlighting a substantial decline in their mental health. As with stress, there was an apparent gradient, with a difference of 7pp between questionable and mild or moderate cognitive impairment that was significant at the 5% level.

In addition to the links between respondents' CDRs and informants' stress and mental health, significant relationships were also found for positive affect and spirituality. Compared to informants of dementia‐free respondents, those of respondents with questionable dementia experienced decreases in the probability of reporting high levels of positive affect and spirituality of 4pp (*p* < .01) and 3.5pp, respectively. As respondents' cognitive impairment progressed to mild or moderate, the reductions in these probabilities became more pronounced, nearing 10pp (*p* < .01) for both outcomes. Thus, for positive affect and spirituality, differences between informants of respondents with questionable dementia and informants of respondents with mild or moderate dementia were not as large as those observed for stress and poor mental health, but still meaningful (> 5pp) and marginally significant (*p* < .10).

The linear regression results, treating the indices of stress, poor mental health, positive affect, and spirituality as continuous dependent variables, aligned with the main findings described above (Table SM3 in the Supplemental Material). Compared to informants of dementia‐free respondents, those of individuals with questionable and mild or moderate dementia experienced increases in the stress index of 0.19 (*p* < .01) and 0.34 (*p* < .01) standard deviations, respectively, with the difference between these coefficients significant at the 5% level. Similarly, their CESD scores increased by 0.16 (*p* < .01) and 0.35 (*p* < .01) standard deviations, with this difference significant at the 1% level. Compared to informants of respondents with no cognitive impairment, those assisting respondents with questionable and mild or moderate impairment experienced decreases in the positive affect index of approximately 0.12 (*p* < .01) and 0.31 (*p* < .01) standard deviations, respectively, with the difference between these two coefficients significant at the 5% level. Similarly, they saw declines in the spirituality index of approximately 0.11 (*p* < .01) and 0.29 (*p* < .01) standard deviations, respectively, with the difference between these two coefficients also significant at the 5% level.

We found notable relationships between informants' characteristics and their well‐being. Below, we highlight a few key findings, with full results provided in Tables SM2 (logit marginal effects) and SM3 (linear regression coefficients) in the Supplemental Material. Compared to the respondents' spouses/partners, sons, daughters, daughters‐in‐law, and other relatives tended to report lower levels of stress and significantly higher levels of positive affect and spirituality. Informants who did not co‐reside with the respondent but had daily contact reported significantly worse well‐being, with higher stress, poorer mental health, and lower positive affect and spirituality compared to those living with the respondent. Crucially, we observed systematic differences in well‐being across caregiving roles. Compared to non‐caregivers, caregivers exhibited a significantly greater probability of reporting above‐median stress levels (ranging from 8pp to 22pp) and above‐median CESD scores (ranging from 3pp to 6pp). Differences in positive outcomes were less pronounced, with only shared caretakers estimated to have lower levels of positive affect relative to non‐caretakers.

### 3.3 Heterogeneous effects of respondents' cognitive impairment by informants' caretaking role

**FIGURE 2:**
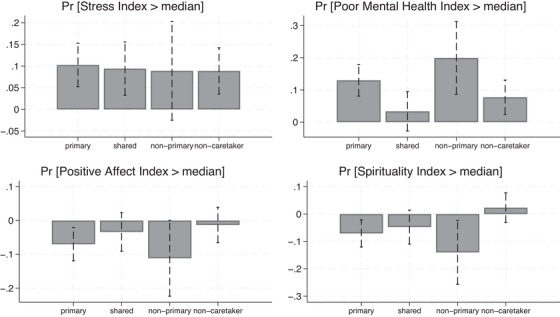
*N* = 4196. The figure reports the average marginal effects (and corresponding 95% confidence interval) of the respondent's probable dementia (CDR > 0) at different informants' caretaking roles (primary, shared, non‐primary caretaker, non‐caretaker). These marginal effects were computed after a Logit regression featuring indicators for the respondent's CDR > 0, indicators for different informants' caretaking roles, and their interactions. All regressions include controls for informants' sex, age, education, relationship with respondent, and frequency of contact with the respondent. The regressions also include controls for respondents' sex, age, rural residence, and number of ADLs. These estimated marginal effects, alongside p‐values testing whether their pairwise comparisons are statistically significant, is in Tables SM4 in the Supplemental Material. ADLs, activities of daily living; CDR, clinical dementia rating.

To explore effect modifications by the informant's caregiving role, we amended our Logit regressions with interactions between indicators for different levels of caregiving responsibility and an indicator for the respondent's probable dementia (CDR > 0). Based on the estimated logit coefficients from these regressions, we computed the marginal effects of the respondent's probable dementia on the probability that informants report high levels (above median) of stress, poor mental health, positive affect, and spirituality, across the four possible caregiving roles elicited by the survey (primary caretaker, shared caretaker, non‐primary caretaker, and non‐caretaker). A visual representation of these marginal effects is provided in Figure 2. Table SM4 in the Supplemental Material reports the same marginal effects, alongside *p* values testing whether their pairwise comparisons are statistically significant.

As shown in the top‐left graph, the negative impact of the respondent's cognitive impairment on the informant's stress was consistently experienced by all informants, regardless of their caregiving role. The increase in the likelihood of reporting high stress associated with the respondent's probable dementia was highest at 10pp for informants who identified as the primary caregiver, but this estimate was not different either in magnitude or statistically from the approximately 9pp increase experienced by non‐caretakers. In contrast, the top‐right graph in Figure 2 reveals heterogeneous effects on mental health. Compared to non‐caretakers, primary caregivers experienced a much more severe impact from the respondent's cognitive impairment. The probability that the informant's CESD score is above the median increased by 13pp among primary caretakers, compared to approximately 8pp among non‐caretakers – a difference that is borderline significant (*p* = .15). Interestingly, the most pronounced effect was observed among non‐primary caregivers, for whom the probability of having an above‐median CESD score rose by 20pp when the respondent had a CDR greater than zero. This increase, however, was not statistically different from the 13pp increase estimated from primary caretakers (*p* = .27). The group for whom the respondent's cognitive impairment had the smallest impact was that of shared caregivers. Among them, the likelihood of exhibiting high levels of poor mental health increased only by 3pp, a non‐statistically significant effect.

Weak evidence of heterogeneous effects was observed for positive affect and spirituality, as shown in the bottom‐left and bottom‐right graphs of Figure 2. When the respondent had probable dementia, primary caregivers saw the probability of reporting high levels of positive affect and spirituality drop by 7pp. In contrast, the respondent's cognitive impairment did not affect these outcomes for non‐caretakers, with estimated marginal effects close to zero. The difference in the impact of the respondent's cognitive impairment between primary caregivers and non‐caretakers was marginally significant for positive affect (*p* = .11) and significant at the 1% level for spirituality. As with mental health, non‐primary caregivers appeared to be the most affected by the respondent's probable dementia. In this group, the estimated reductions in the likelihood of reporting high positive affect and spirituality were 11pp (marginally different from that of non‐caretakers, *p* = .12) and 14pp (statistically different from that of non‐caretakers, *p* < .05), respectively. Although the estimated effects of respondents' cognitive impairment on positive affect and spirituality were larger for non‐primary caregivers than for primary caregivers, the differences were not statistically significant.

We observed similar patterns when estimating marginal effects using linear regressions, treating the indices of stress, poor mental health, positive affect, and spirituality as continuous dependent variables (Table SM5 in the Supplemental Material). However, in this case, the greater variability of the outcome variables improved the precision of the estimated coefficients. As a result, differences between caregiving groups that were only borderline significant in the logit analysis became significant at either the 10% or 5% level.

## 4 DISCUSSION

In a first‐in‐kind analysis of newly collected data from the LASI‐DAD, we investigated the relationship between cognitive impairment in individuals and the well‐being of their caregivers in India. Specifically, we examined how cognitive impairment in LASI‐DAD respondents impacted the emotional and psychological state of informants, who were primarily family members, and how these effects varied based on their caregiving roles.

Our findings supported the hypothesis that respondents' cognitive impairment was linked to reduced well‐being among informants, contributing new empirical evidence from the Indian context to complement previous research from Western countries. Assisting individuals with cognitive impairment and dementia places considerable strain on caregivers. Informants caring for respondents with mild to moderate dementia reported higher perceived stress and worse mental health. Additionally, they showed reductions in positive affect and spirituality, highlighting that dementia caregiving affects not only stress and mental health but also one's sense of happiness and inner peace.

We also found evidence supporting our second hypothesis, which proposed that the impact of respondents' cognitive impairment on informants' well‐being would differ based on caregiving roles and be more pronounced for those with greater caregiving responsibility. Compared to non‐caregivers, primary caregivers – who took on full responsibility for respondents' care – experienced worse mental health and lower positive affect and spirituality. However, non‐primary caregivers also showed significant declines in well‐being, particularly in terms of poorer mental health and reduced positive affect and spirituality. To better understand the latter finding, we investigated differences in relationship types and frequency of contact with the respondent across informant groups. A key distinction – illustrated in Figure SM3 in the Supplemental Material – is that non‐primary caregivers are less likely to be spouses/partners of the care recipient and more likely have other types of relationships, including grandchildren (60%), other relative (20%), sibling (12%), son‐in‐law (3%), friend (3%), and servant (2%). Additionally, and plausibly because of their relationship with the care recipient, they are significantly less likely to co‐reside with the care recipient (62% vs > 80% for other caregivers and non‐caregivers). Nevertheless, most still maintain daily contacts with the care recipient (Figure SM4 in the Supplemental Material). These differences in group composition led to the following considerations. On one hand, co‐residing non‐primary caregivers may experience indirect burdens, such as disruptions in family dynamics, increased household responsibilities, or the emotional strain of witnessing cognitive decline. Despite being less directly involved in caregiving, the implicit expectations to contribute to care, coupled with their relatively younger age (average age is the lowest among non‐primary caretakers at 40), may make them particularly vulnerable to the negative well‐being effects of dementia caregiving. On the other hand, non‐primary caregivers who do not co‐reside face a different but equally significant burden, shaped by travel demands, disruptions to daily life, logistical challenges, the emotional toll of distance, and a diminished sense of control over caregiving tasks. The need to frequently visit the care recipient while balancing other responsibilities may exacerbate their stress and negatively impact their overall well‐being.

The empirical evidence documented in this study suggests that, although caring for an elderly person in India is seldom perceived or recognized as a burden^17^, ^18^ and awareness of cognitive impairment is limited,^19^ assisting a person with dementia extends far beyond providing physical support. It significantly impacts the emotional and spiritual well‐being of family members. This underscores the substantial psychological challenges caregivers face, which often remain unspoken and unacknowledged.

Our results must be understood in the cultural context of India, where caregiving for elderly family members is predominantly a familial obligation, usually falling on women.^20^ The absence of a robust formal care infrastructure for the elderly places additional pressure on families to provide care for individuals with dementia or cognitive decline. Consequently, caregivers may experience increased stress and mental health issues, which are compounded by the lack of professional support systems such as counseling or respite care. Another critical cultural factor is the social stigma associated with mental health, which may deter caregivers from seeking help.^21^ In many cases, caregivers may prioritize the needs of the care recipient over their own, leading to burnout and emotional exhaustion. Furthermore, the joint family structure, common in India, can either mitigate or exacerbate caregiving challenges. While living in a joint family may provide additional hands to share caregiving duties, it can also lead to conflicts over caregiving responsibilities, particularly in cases where expectations are gendered. Moreover, living with a cognitively impaired individual may negatively impact the well‐being of household members who are not directly involved in caregiving. This may be particularly true if their limited caregiving experience leaves them less equipped to handle disruptive behaviors or unexpected situations that arise from the respondent's cognitive impairment. Spirituality, a significant aspect of Indian culture, also plays a complex role in caregiving. While spirituality may offer a coping mechanism for some caregivers, providing a sense of purpose and resilience, our analysis suggests that assisting a dementia patient can erode this spiritual support, particularly among those who bear the brunt of caregiving duties.

A key strength of this study was the development of a caregiving module within the LASI‐DAD informant questionnaire, allowing us to collect detailed data on informants' caregiving responsibilities and well‐being. By integrating this with LASI‐DAD respondents' cognitive assessments and dementia diagnoses, we examined how cognitive impairment affected caregiver well‐being in India for the first time. Another strength is the focus on a culturally relevant population. By examining caregiving in India, where family‐based care is the norm and happens in large, multigenerational households, this study contributes to a growing body of research on caregiving in LMICs, highlighting the unique challenges caregivers face in these settings. Additionally, including positive affect and spirituality offers a more holistic view of caregiver well‐being beyond stress and mental health.

Clearly, this study has limitations. First, the cross‐sectional nature of the data limits our ability to draw causal inferences. While the analysis reveals significant associations, longitudinal data are needed to examine how these relationships evolve over time as cognitive impairment progresses. As LASI‐DAD will follow caregivers and care recipients over time, prospective data will provide valuable insights into the long‐term effects of caregiving on well‐being. Second, while the study accounts for various caregiver characteristics (such as gender, relationship to the respondent, and frequency of contact), it lacks data on broader social and economic factors, like financial strain and barriers to labor force participation, which may impact caregiver well‐being, especially in low‐income settings like India. Finally, self‐reported well‐being may introduce bias, as caregivers might underreport stress and poor mental health due to stigma and social desirability. This likely underestimates the true effects, so the estimated strong associations between cognitive impairment and reduced caregiver well‐being suggest the actual challenges of caregiving in India may be even greater.

This study highlighted the significant emotional, psychological, and spiritual toll that caregiving for individuals with cognitive impairment takes on family members in India. The findings underscored the need for greater support for caregivers in terms of formal care infrastructure and mental health resources. As India's population continues to age, the demand for caregiving will only increase, making it crucial to address the well‐being of caregivers to ensure sustainable caregiving practices. This study enhances our understanding of caregiving in LMICs by situating findings in India's cultural context. It sets the stage for future research using representative caregiver and care recipient data. The global aging study network, of which LASI‐DAD is a part, offers a platform for comparing caregiving experiences across countries.^22^

## CONFLICT OF INTEREST STATEMENT

All authors have no conflicts of interest. Author disclosures are available in the Supporting Information.

## CONSENT STATEMENT

Written consent was obtained from each participating respondent and informant in the form of signature or thumb print. The LASI‐DAD study received Institutional Review Board approval at the University of Southern California (UP‐15‐00684) and the Indian Council of Medical Research (2022‐16741).

## Supporting information



Supporting Information

Supporting Information
