# Supplementary material for: Dementia caregiving in India: New evidence from a National representative sample
Source: Alzheimers Dement. 2025 May 15;21(5):e70266. doi: 10.1002/alz.70266 (PMC12079535; doi:10.1002/alz.70266)

**Dementia Caregiving in India: New Evidence from a National Representative Sample**

**Supplemental Material**

**Table SM1: Respondents’ Characteristics by Informants’ Caretaking Role**

|  | Informant’s Role as R’s caretaker | | | |
| --- | --- | --- | --- | --- |
|  | **Primary** | **Shared** | **Non-primary** | **Non-caretaker** |
| Sex: perc (sd) | | | | |
| Male | 56.10% (49.64) | 55.42% (49.73) | 58.15% (49.42) | 60.66% (48.87) |
| Female | 43.90% (49.64) | 44.58% (49.73) | 41.85% (49.42) | 39.34% (48.87) |
| Age: perc (sd) | | | | |
| 60-64 | 13.49% (34.17) | 9.86% (29.83) | 13.33% (34.06) | 17.52% (38.03) |
| 65-69 | 28.99% (45.39) | 25.74% (43.74) | 26.67% (44.30) | 27.15% (44.49) |
| 70-74 | 24.19% (42.84) | 27.61% (44.73) | 23.33% (42.37) | 25.99% (43.87) |
| 75-79 | 16.34% (36.99) | 18.93% (39.20) | 17.41% (37.99) | 15.33% (36.04) |
| 80+ | 16.99% (37.57) | 17.85% (38.31) | 19.26% (39.51) | 14.01% (34.73) |
| Residence: perc (sd) | | | | |
| Urban | 31.78% (46.58) | 20.51% (40.40) | 26.67% (44.30) | 36.42% (48.14) |
| Rural | 68.22% (46.58) | 79.49% (40.40) | 73.33% (44.30) | 63.58% (48.14) |
| Cognition: mean (sd) | | | | |
| Total Z-Score | -0.09 (0.86) | -0.28 (0.83) | -0.20 (0.88) | -0.01 (0.82) |
| CDR: perc (sd) | | | | |
| No impairment | 48.12% (49.98) | 44.08% (49.67) | 46.67% (49.98) | 59.78% (49.05) |
| Questionable impairment | 46.56% (49.90) | 49.01% (50.01) | 45.93% (49.93) | 38.69% (48.72) |
| Mild impairment | 4.99% (21.79) | 6.51% (24.68) | 7.41% (26.24) | 1.46% (12.00) |
| Moderate impairment | 0.32% (5.69) | 0.39% (6.27) | 0.00% | 0.07% (2.70) |
| Sever impairment | 0.00% | 0.00% | 0.00% | 0.00% |
| ADLs: mean (sd) | | | | |
| Number | 1.13 (1.75) | 1.16 (1.66) | 1.07 (1.63) | 1.00 (1.48) |

**Figure SM1: Total Cognition Score by Informants' Caretaking Role**


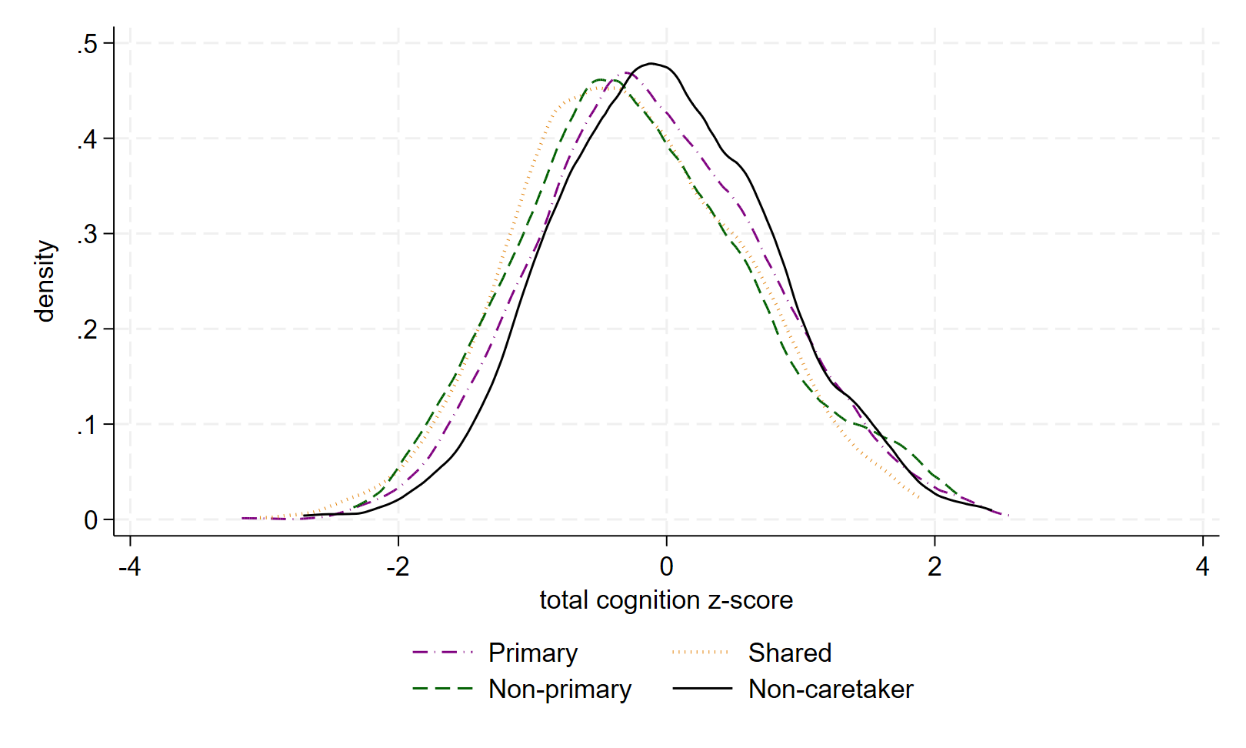


**Figure SM2: Prevalence of ADLs by Informants' Caretaking Role**


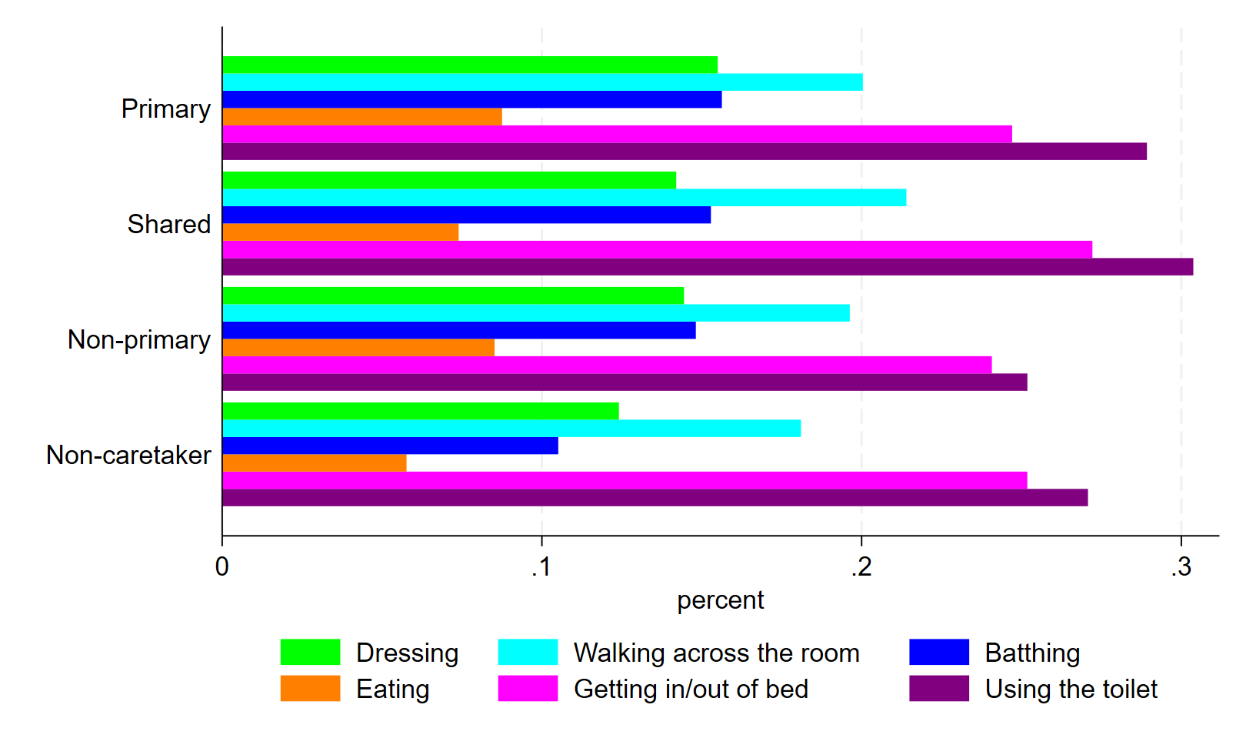


**Table SM2: Logit Marginal Effects – Informants’ Indexes > Median**

|  | Stress | Poor Mental Health | Positive Affect | Spirituality |
| --- | --- | --- | --- | --- |
|  |  |  |  |  |
| Inf: male | -0.066** | -0.127*** | 0.047* | 0.012 |
|  | (0.026) | (0.026) | (0.026) | (0.027) |
| Inf: age 30-39 | -0.032 | -0.036 | 0.011 | 0.030 |
|  | (0.025) | (0.025) | (0.023) | (0.025) |
| Inf: age 40-49 | -0.050* | 0.025 | 0.005 | 0.024 |
|  | (0.027) | (0.027) | (0.025) | (0.027) |
| Inf: age 50-59 | -0.060* | -0.027 | 0.079*** | 0.044 |
|  | (0.032) | (0.032) | (0.031) | (0.032) |
| Inf: age 60-69 | -0.039 | 0.064* | 0.042 | 0.045 |
|  | (0.036) | (0.036) | (0.035) | (0.037) |
| Inf: age 60+ | -0.028 | 0.060 | 0.097** | 0.073* |
|  | (0.041) | (0.041) | (0.041) | (0.042) |
| Inf: education less than secondary | -0.016 | -0.037* | 0.075*** | 0.072*** |
|  | (0.022) | (0.021) | (0.020) | (0.021) |
| Inf: education secondary or higher | -0.124*** | -0.111*** | 0.126*** | 0.091*** |
|  | (0.021) | (0.021) | (0.020) | (0.021) |
| Inf: son | -0.024 | -0.003 | 0.062* | 0.069* |
|  | (0.038) | (0.038) | (0.035) | (0.038) |
| Inf: daughter | -0.035 | -0.001 | 0.084** | 0.008 |
|  | (0.041) | (0.040) | (0.039) | (0.040) |
| Inf: daughter-in-law | -0.055 | 0.006 | 0.090*** | 0.082** |
|  | (0.036) | (0.036) | (0.034) | (0.036) |
| Inf: other | -0.100*** | -0.017 | 0.136*** | 0.141*** |
|  | (0.037) | (0.037) | (0.035) | (0.037) |
| Inf: daily contact | 0.087*** | 0.076*** | -0.041* | -0.115*** |
|  | (0.023) | (0.023) | (0.022) | (0.022) |
| Inf: several times a week or less | -0.011 | 0.014 | -0.025 | -0.018 |
|  | (0.041) | (0.041) | (0.039) | (0.043) |
| Inf: primary caretaker | 0.092*** | 0.029 | 0.023 | 0.013 |
|  | (0.018) | (0.018) | (0.018) | (0.019) |
| Inf: shared caretaker | 0.083*** | 0.053** | -0.060*** | 0.000 |
|  | (0.021) | (0.021) | (0.020) | (0.021) |
| Inf: non-primary caretaker | 0.228*** | 0.061* | -0.031 | -0.054 |
|  | (0.032) | (0.032) | (0.032) | (0.033) |
| R: male | -0.014 | -0.014 | 0.031* | 0.028 |
|  | (0.017) | (0.017) | (0.017) | (0.017) |
| R: age 65-69 | 0.038 | -0.012 | -0.031 | -0.030 |
|  | (0.025) | (0.025) | (0.024) | (0.025) |
| R: age 70-74 | 0.017 | -0.014 | -0.007 | -0.015 |
|  | (0.025) | (0.025) | (0.025) | (0.026) |
| R: age 75-79 | 0.006 | -0.012 | -0.038 | -0.036 |
|  | (0.028) | (0.028) | (0.028) | (0.029) |
| R: age 80+ | 0.016 | -0.031 | -0.040 | -0.062** |
|  | (0.029) | (0.029) | (0.028) | (0.030) |
| R: rural residence | 0.032* | 0.015 | -0.013 | -0.007 |
|  | (0.017) | (0.017) | (0.017) | (0.017) |
| R: number of ADLs | 0.009** | 0.019*** | -0.011** | -0.005 |
|  | (0.005) | (0.005) | (0.005) | (0.005) |
|  |  |  |  |  |
| CDR: questionable impairment | 0.090*** | 0.089*** | -0.042*** | -0.035** |
|  | (0.016) | (0.016) | (0.016) | (0.016) |
| CDR: mild/moderate impairment | 0.163*** | 0.165*** | -0.096*** | -0.100*** |
|  | (0.038) | (0.038) | (0.035) | (0.038) |
|  |  |  |  |  |
| Observations | 4,196 | 4,196 | 4,196 | 4,196 |
| Pseudo R-squared | 0.043 | 0.044 | 0.023 | 0.017 |

Robust standard errors in parentheses *** p<0.01, ** p<0.05, * p<0.1

**Table SM3: Linear Regression Coefficients – Informants’ Indexes as Continuous Variables**

|  | Stress | Poor Mental Health | Positive Affect | Spirituality |
| --- | --- | --- | --- | --- |
|  |  |  |  |  |
| Inf: male | -0.113** | -0.295*** | 0.139*** | 0.123** |
|  | (0.053) | (0.052) | (0.053) | (0.055) |
| Inf: age 30-39 | -0.069 | -0.068 | 0.068 | 0.017 |
|  | (0.048) | (0.048) | (0.049) | (0.049) |
| Inf: age 40-49 | -0.095* | 0.028 | 0.042 | 0.006 |
|  | (0.051) | (0.051) | (0.051) | (0.052) |
| Inf: age 50-59 | -0.128** | -0.082 | 0.141** | 0.078 |
|  | (0.062) | (0.063) | (0.063) | (0.065) |
| Inf: age 60-69 | -0.193*** | 0.058 | 0.123* | 0.051 |
|  | (0.070) | (0.072) | (0.071) | (0.075) |
| Inf: age 60+ | -0.173** | 0.024 | 0.172** | 0.131 |
|  | (0.078) | (0.079) | (0.082) | (0.085) |
| Inf: education less than secondary | -0.093** | -0.079* | 0.131*** | 0.106** |
|  | (0.042) | (0.041) | (0.043) | (0.043) |
| Inf: education secondary or higher | -0.295*** | -0.278*** | 0.254*** | 0.151*** |
|  | (0.042) | (0.041) | (0.042) | (0.043) |
| Inf: son | -0.149** | -0.084 | 0.181** | 0.159** |
|  | (0.075) | (0.076) | (0.076) | (0.080) |
| Inf: daughter | -0.184** | -0.082 | 0.166** | 0.119 |
|  | (0.079) | (0.084) | (0.084) | (0.084) |
| Inf: daughter-in-law | -0.182*** | -0.063 | 0.243*** | 0.242*** |
|  | (0.071) | (0.073) | (0.073) | (0.074) |
| Inf: other | -0.242*** | -0.154** | 0.354*** | 0.280*** |
|  | (0.070) | (0.072) | (0.073) | (0.076) |
| Inf: daily contact | 0.137*** | 0.075* | -0.134*** | -0.195*** |
|  | (0.043) | (0.043) | (0.045) | (0.044) |
| Inf: several times a week or less | 0.042 | -0.069 | -0.069 | -0.039 |
|  | (0.075) | (0.075) | (0.084) | (0.086) |
| Inf: primary caretaker | 0.242*** | 0.066* | 0.054 | 0.023 |
|  | (0.038) | (0.037) | (0.037) | (0.038) |
| Inf: shared caretaker | 0.211*** | 0.135*** | -0.150*** | 0.031 |
|  | (0.042) | (0.042) | (0.042) | (0.042) |
| Inf: non-primary caretaker | 0.520*** | 0.145** | -0.034 | -0.141** |
|  | (0.065) | (0.061) | (0.065) | (0.069) |
| R: male | -0.039 | -0.024 | 0.046 | 0.083** |
|  | (0.033) | (0.033) | (0.034) | (0.034) |
| R: age 65-69 | 0.121** | 0.051 | -0.076 | -0.056 |
|  | (0.050) | (0.049) | (0.049) | (0.051) |
| R: age 70-74 | 0.042 | 0.023 | -0.037 | -0.018 |
|  | (0.052) | (0.050) | (0.051) | (0.053) |
| R: age 75-79 | 0.070 | 0.058 | -0.061 | -0.080 |
|  | (0.056) | (0.054) | (0.056) | (0.058) |
| R: age 80+ | 0.041 | 0.007 | -0.122** | -0.103* |
|  | (0.059) | (0.056) | (0.059) | (0.059) |
| R: rural residence | 0.048 | 0.007 | -0.039 | -0.002 |
|  | (0.035) | (0.035) | (0.035) | (0.035) |
| R: number of ADLs | 0.021** | 0.053*** | -0.037*** | -0.012 |
|  | (0.009) | (0.010) | (0.010) | (0.010) |
|  |  |  |  |  |
| CDR: questionable impairment | 0.191*** | 0.165*** | -0.126*** | -0.108*** |
|  | (0.032) | (0.032) | (0.032) | (0.032) |
| CDR: mild/moderate impairment | 0.342*** | 0.348*** | -0.307*** | -0.286*** |
|  | (0.073) | (0.071) | (0.076) | (0.076) |
|  |  |  |  |  |
| Constant | 0.043 | 0.095 | -0.250*** | -0.236** |
|  | (0.091) | (0.092) | (0.093) | (0.095) |
|  |  |  |  |  |
| Observations | 4,196 | 4,196 | 4,196 | 4,196 |
| R-squared | 0.068 | 0.077 | 0.046 | 0.027 |

Robust standard errors in parentheses *** p<0.01, ** p<0.05, * p<0.1

**Table SM4: Marginal Effects of Respondents’ Cognitive Impairment**

**on Informants’ Outcomes by Informants’ Caretaking Role**

(Dependent Variable: indicator for Index > Median)

|  | Stress | Poor Mental Health | Positive Affect | Spirituality |
| --- | --- | --- | --- | --- |
|  |  |  |  |  |
| (1) Primary caretaker | 0.103*** | 0.130*** | -0.070*** | -0.071*** |
|  | (0.026) | (0.025) | (0.025) | (0.025) |
| (2) Shared caretaker | 0.094*** | 0.034 | -0.034 | -0.048 |
|  | (0.031) | (0.031) | (0.029) | (0.032) |
| (3) Non-primary caretaker | 0.089 | 0.199*** | -0.112* | -0.140** |
|  | (0.058) | (0.058) | (0.057) | (0.059) |
| (4) Non-caretaker | 0.089*** | 0.077*** | -0.013 | 0.023 |
|  | (0.027) | (0.027) | (0.027) | (0.028) |
| Null Hypothesis (p-value) |  |  |  |  |
| H_0_: (1)=(2) | 0.833 | 0.014 | 0.339 | 0.562 |
| H_0_: (1)=(3) | 0.828 | 0.267 | 0.504 | 0.281 |
| H_0_: (1)=(4) | 0.707 | 0.149 | 0.115 | 0.011 |
| H_0_: (2)=(3) | 0.936 | 0.011 | 0.223 | 0.168 |
| H_0_: (2)=(4) | 0.894 | 0.286 | 0.599 | 0.089 |
| H_0_: (3)=(4) | 0.997 | 0.054 | 0.117 | 0.012 |

Robust standard errors in parentheses *** p<0.01, ** p<0.05, * p<0.1.

Average marginal effects of the respondent’s probable dementia (CDR>0) at different informants’ caretaking roles (primary, shared, non-primary caretaker, non-caretaker). These marginal effects were computed after a Logit regression featuring indicators for the respondent’s CDR>0, indicators for different informants’ caretaking roles, and their interactions. All regressions also included controls for informants’ sex, age, education, relationship with respondent, and frequency of contact with the respondent, as well as the respondent’s sex, age, rural residence, and number of ADLs.

**Table SM5: Marginal Effects of Respondents’ Cognitive Impairment**

**on Informants’ Outcomes by Informants’ Caretaking Role**

(Dependent Variable: index as continuous variable)

|  | Stress | Poor Mental Health | Positive Affect | Spirituality |
| --- | --- | --- | --- | --- |
|  |  |  |  |  |
| (1) Primary caretaker | 0.230*** | 0.272*** | -0.201*** | -0.195*** |
|  | (0.049) | (0.049) | (0.050) | (0.051) |
| (2) Shared caretaker | 0.178*** | 0.057 | -0.098 | -0.087 |
|  | (0.060) | (0.061) | (0.061) | (0.060) |
| (3) Non-primary caretaker | 0.194* | 0.343*** | -0.355*** | -0.442*** |
|  | (0.117) | (0.106) | (0.117) | (0.125) |
| (4) Non-caretaker | 0.188*** | 0.127** | -0.051 | 0.007 |
|  | (0.058) | (0.057) | (0.056) | (0.057) |
| Null Hypothesis (p-value) |  |  |  |  |
| H_0_: (1)=(2) | 0.503 | 0.005 | 0.190 | 0.162 |
| H_0_: (1)=(3) | 0.779 | 0.538 | 0.224 | 0.065 |
| H_0_: (1)=(4) | 0.575 | 0.050 | 0.042 | 0.007 |
| H_0_: (2)=(3) | 0.904 | 0.019 | 0.051 | 0.009 |
| H_0_: (2)=(4) | 0.908 | 0.396 | 0.567 | 0.250 |
| H_0_: (3)=(4) | 0.961 | 0.072 | 0.018 | 0.001 |

Robust standard errors in parentheses *** p<0.01, ** p<0.05, * p<0.1.

Average marginal effects of the respondent’s probable dementia (CDR>0) at different informants’ caretaking roles (primary, shared, non-primary caretaker, non-caretaker). These marginal effects were computed after a linear regression featuring indicators for the respondent’s CDR>0, indicators for different informants’ caretaking roles, and their interactions. All regressions also included controls for informants’ sex, age, education, relationship with respondent, and frequency of contact with the respondent, as well as the respondent’s sex, age, rural residence, and number of ADLs.

**Figure SM3: Relationship with the Respondent by Informants' Caretaking Role**


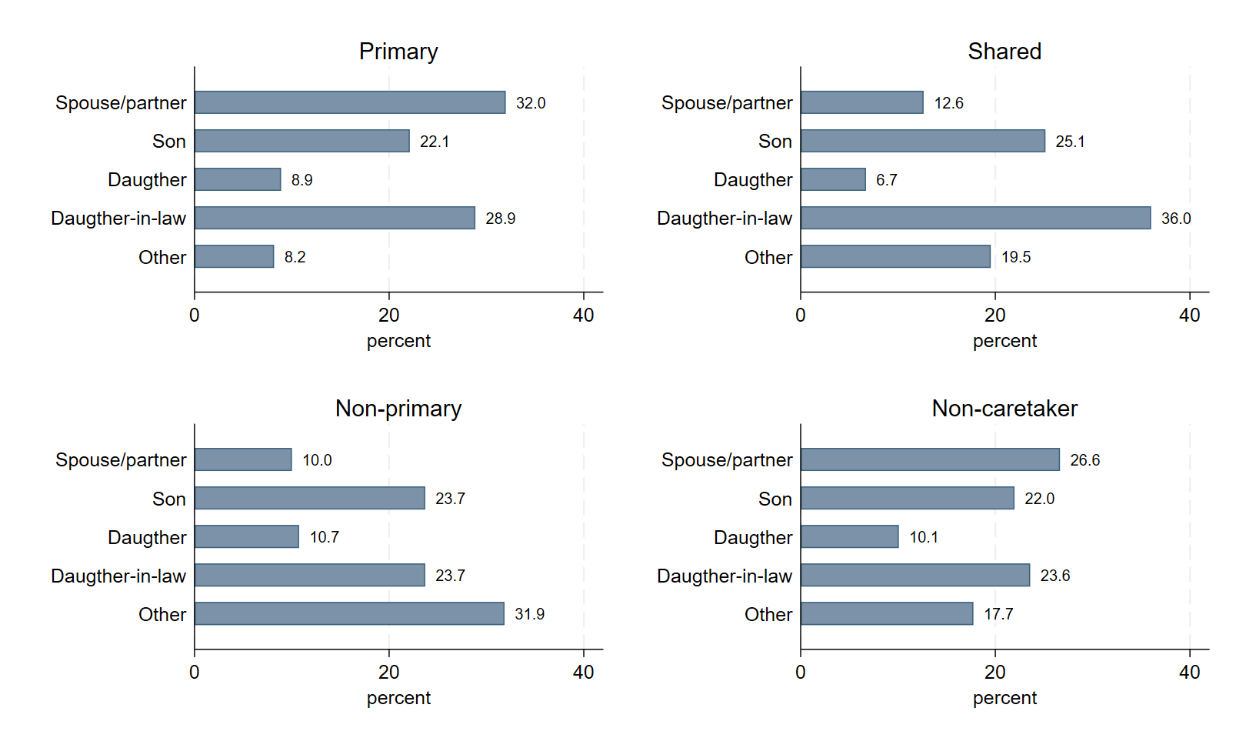


**Figure SM4: Frequency of Contacts with the Respondent by Informants' Caretaking Role**


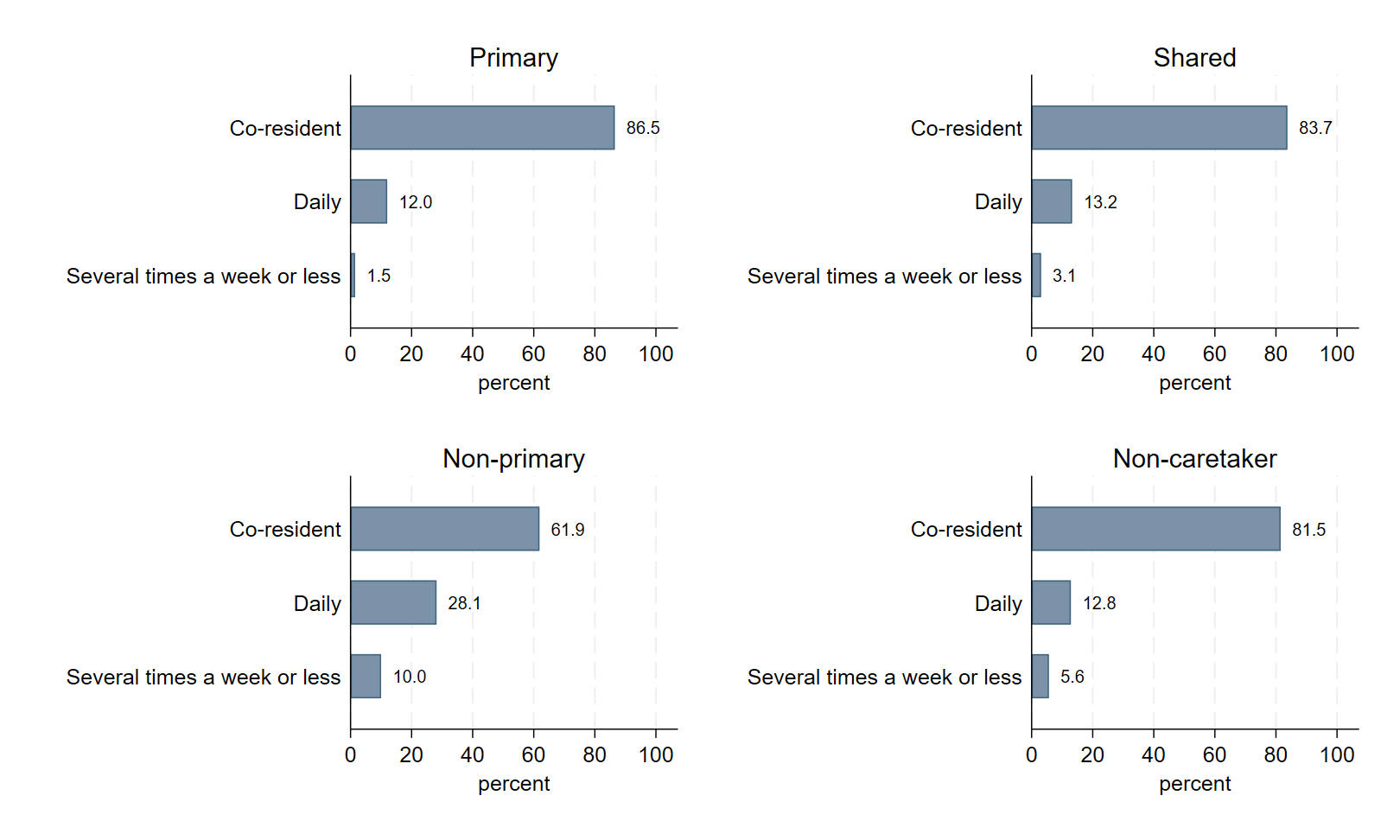

Supplement: Supplementary file 1 — Supporting Information [file ALZ-21-e70266-s001.docx]
