# Supplementary material for: Dementia caregiving in India: New evidence from a National representative sample
Source: Alzheimers Dement. 2025 May 15;21(5):e70266. doi: 10.1002/alz.70266 (PMC12079535; doi:10.1002/alz.70266)
Supplement: Supplementary file 2 — Supporting Information [file ALZ-21-e70266-s002.pdf]

ICMJE DISCLOSURE FORM

Date:___3/11/2025____
Your Name:__Marco Angrisani____
Manuscript Title:_ Dementia Caregiving in India: New Evidence from a National Representative Sample
Manuscript number (if known):____

In the interest of transparency, we ask you to disclose all relationships/activities/interests listed below that are related to the content of your manuscript. "Related" means any relation with for-profit or not-for-profit third parties whose interests may be affected by the content of the manuscript. Disclosure represents a commitment to transparency and does not necessarily indicate a bias. If you are in doubt about whether to list a relationship/activity/interest, it is preferable that you do so.

The following questions apply to the author's relationships/activities/interests as they relate to the current manuscript only.

The author's relationships/activities/interests should be defined broadly. For example, if your manuscript pertains to the epidemiology of hypertension, you should declare all relationships with manufacturers of antihypertensive medication, even if that medication is not mentioned in the manuscript.

In item #1 below, report all support for the work reported in this manuscript without time limit. For all other items, the time frame for disclosure is the past 36 months.

		**Name all entities with whom you have this relationship or indicate none (add rows as needed)**	**Specifications/Comments (e.g., if payments were made to you or to your institution)**
		**Time frame: Since the initial planning of the work**	
1	All support for the present manuscript (e.g., funding, provision of study materials, medical writing, article processing charges, etc.) **No time limit for this item.**	National Institute on Aging (R01AG051125 U01AG058499 RF1AG055273)	
		**Time frame: past 36 months**	
2	Grants or contracts from any entity (if not indicated in item #1 above).	____None	National Institute on Aging (R01AG022481, R01AG068190, 1R01AG077280)
3	Royalties or licenses	_X___None	

4	Consulting fees	__X__None	
5	Payment or honoraria for lectures, presentations, speakers bureaus, manuscript writing or educational events	__X__None	
6	Payment for expert testimony	__X__None	
7	Support for attending meetings and/or travel	__X__None	
8	Patents planned, issued or pending	__X__None	
9	Participation on a Data Safety Monitoring Board or Advisory Board	____None	Add Health
10	Leadership or fiduciary role in other board, society, committee or advocacy group, paid or unpaid	__X__None	
11	Stock or stock options	__X__None	
12	Receipt of equipment, materials, drugs, medical writing, gifts or other services	__X__None	
13	Other financial or non-financial interests	__X__None	

Please place an "X" next to the following statement to indicate your agreement:

__X_ I certify that I have answered every question and have not altered the wording of any of the questions on this form.

ICMJE DISCLOSURE FORM

Date:___3/11/2025____
Your Name:__ Nicholas S. Reed ____
Manuscript Title:_ Dementia Caregiving in India: New Evidence from a National Representative Sample
Manuscript number (if known):____

In the interest of transparency, we ask you to disclose all relationships/activities/interests listed below that are related to the content of your manuscript. "Related" means any relation with for-profit or not-for-profit third parties whose interests may be affected by the content of the manuscript. Disclosure represents a commitment to transparency and does not necessarily indicate a bias. If you are in doubt about whether to list a relationship/activity/interest, it is preferable that you do so.

The following questions apply to the author's relationships/activities/interests as they relate to the current manuscript only.

The author's relationships/activities/interests should be defined broadly. For example, if your manuscript pertains to the epidemiology of hypertension, you should declare all relationships with manufacturers of antihypertensive medication, even if that medication is not mentioned in the manuscript.

In item #1 below, report all support for the work reported in this manuscript without time limit. For all other items, the time frame for disclosure is the past 36 months.

		**Name all entities with whom you have this relationship or indicate none (add rows as needed)**	**Specifications/Comments (e.g., if payments were made to you or to your institution)**
**Time frame: Since the initial planning of the work**			
1	All support for the present manuscript (e.g., funding, provision of study materials, medical writing, article processing charges, etc.) **No time limit for this item.**	National Institute on Aging (R01AG051125 U01AG058499 RF1AG055273)	
		National Institute on Aging (K23AG065443)	Funds 75% general effort of Nicholas Reed
**Time frame: past 36 months**			
2	Grants or contracts from any entity (if not indicated in item #1 above).	____None	National Institute on Aging (R01AG022481, R01AG068190, 1R01AG077280)
			National Institute on Aging (K23AG065443)
3	Royalties or licenses	_X___None	

4	Consulting fees	__X__None	
5	Payment or honoraria for lectures, presentations, speakers bureaus, manuscript writing or educational events	__X__None	
6	Payment for expert testimony	__X__None	
7	Support for attending meetings and/or travel	__X__None	
8	Patents planned, issued or pending	__X__None	
9	Participation on a Data Safety Monitoring Board or Advisory Board	__X__None	
10	Leadership or fiduciary role in other board, society, committee or advocacy group, paid or unpaid	__ __None	Neosensory Inc, Scientific Advisory Board 2021-2023 (Reed)
11	Stock or stock options	__X__None	
12	Receipt of equipment, materials, drugs, medical writing, gifts or other services	__X__None	
13	Other financial or non-financial interests	__X__None	

Please place an "X" next to the following statement to indicate your agreement:

__X_ I certify that I have answered every question and have not altered the wording of any of the questions on this form.

ICMJE DISCLOSURE FORM

Date:___3/11/2025____
Your Name:__ Joyita Banerjee ____
Manuscript Title:_ Dementia Caregiving in India: New Evidence from a National Representative Sample
Manuscript number (if known):____

In the interest of transparency, we ask you to disclose all relationships/activities/interests listed below that are related to the content of your manuscript. "Related" means any relation with for-profit or not-for-profit third parties whose interests may be affected by the content of the manuscript. Disclosure represents a commitment to transparency and does not necessarily indicate a bias. If you are in doubt about whether to list a relationship/activity/interest, it is preferable that you do so.

The following questions apply to the author's relationships/activities/interests as they relate to the current manuscript only.

The author's relationships/activities/interests should be defined broadly. For example, if your manuscript pertains to the epidemiology of hypertension, you should declare all relationships with manufacturers of antihypertensive medication, even if that medication is not mentioned in the manuscript.

In item #1 below, report all support for the work reported in this manuscript without time limit. For all other items, the time frame for disclosure is the past 36 months.

		Name all entities with whom you have this relationship or indicate none (add rows as needed)	Specifications/Comments (e.g., if payments were made to you or to your institution)
**Time frame: Since the initial planning of the work**			
1	All support for the present manuscript (e.g., funding, provision of study materials, medical writing, article processing charges, etc.) **No time limit for this item.**	National Institute on Aging (R01AG051125 U01AG058499 RF1AG055273)	
**Time frame: past 36 months**			
2	Grants or contracts from any entity (if not indicated in item #1 above).	____None	National Institute on Aging (R01AG022481, R01AG068190, 1R01AG077280)
3	Royalties or licenses	_X___None	

4	Consulting fees	__X__None	
5	Payment or honoraria for lectures, presentations, speakers bureaus, manuscript writing or educational events	__X__None	
6	Payment for expert testimony	__X__None	
7	Support for attending meetings and/or travel	__X__None	
8	Patents planned, issued or pending	__X__None	
9	Participation on a Data Safety Monitoring Board or Advisory Board	__X__None	
10	Leadership or fiduciary role in other board, society, committee or advocacy group, paid or unpaid	__X__None	
11	Stock or stock options	__X__None	
12	Receipt of equipment, materials, drugs, medical writing, gifts or other services	__X__None	
13	Other financial or non-financial interests	__X__None	

Please place an "X" next to the following statement to indicate your agreement:

__X_ I certify that I have answered every question and have not altered the wording of any of the questions on this form.

ICMJE DISCLOSURE FORM

Date:____________3/11/2025____
Your Name:_______Jinkook Lee____
Manuscript Title:_ Dementia Caregiving in India: New Evidence from a National Representative Sample
Manuscript number (if known):____

In the interest of transparency, we ask you to disclose all relationships/activities/interests listed below that are related to the content of your manuscript. "Related" means any relation with for-profit or not-for-profit third parties whose interests may be affected by the content of the manuscript. Disclosure represents a commitment to transparency and does not necessarily indicate a bias. If you are in doubt about whether to list a relationship/activity/interest, it is preferable that you do so.

The following questions apply to the author's relationships/activities/interests as they relate to the current manuscript only.

The author's relationships/activities/interests should be defined broadly. For example, if your manuscript pertains to the epidemiology of hypertension, you should declare all relationships with manufacturers of antihypertensive medication, even if that medication is not mentioned in the manuscript.

In item #1 below, report all support for the work reported in this manuscript without time limit. For all other items, the time frame for disclosure is the past 36 months.

		Name all entities with whom you have this relationship or indicate none (add rows as needed)	Specifications/Comments (e.g., if payments were made to you or to your institution)
**Time frame: Since the initial planning of the work**			
1	All support for the present manuscript (e.g., funding, provision of study materials, medical writing, article processing charges, etc.) **No time limit for this item.**	National Institute on Aging (R01AG051125 U01AG058499 RF1AG055273)	
**Time frame: past 36 months**			
2	Grants or contracts from any entity (if not indicated in item #1 above).	____None	National Institute on Aging (R01AG070953, R01AG070953, P30AG17265, R01AG042778, R01AG030153, U24AG072699, R24AG048024, R01AG077001, R56AG056117, U01AG054590-04S2, RF1AG055654
			BrightFocus Bold Ideas Initiative Program CA2023001

3	Royalties or licenses	_x___None	
4	Consulting fees	____None	RAND Corporation
5	Payment or honoraria for lectures, presentations, speakers bureaus, manuscript writing or educational events	____None	National Institute on Health
			University of California, Berkely
			Southern Illinois University School of Medicine
6	Payment for expert testimony	_x___None	
7	Support for attending meetings and/or travel	____None	Venice International University
		World Health Organization	University College London
			Davos Alzheimer's Collaboratory
8	Patents planned, issued or pending	x____None	
9	Participation on a Data Safety Monitoring Board or Advisory Board	____None	English Longitudinal Study of Ageing, China Health and Retirement Longitudinal Study, Japanese Study of Aging and Retirement
		Asian Development Bank	Chitwan Valley Family Study-Study of Cognition and Aging in Nepal, Lausanne Cohort 65+ study, AL-SEHA
		WHO Consortium of Metrics and Evidence for Healthy Ageing	Berkely Initiative for Transparency in the Social Sciences, Malawi Longitudinal Study of Families and Health, Longitudinal Study of Health and Ageing in Kenya, RCT: Cognitive Level Enhancement through Vision Exams and Refraction, Malaysia Ageing and Retirement Study
10	Leadership or fiduciary role in other board, society, committee or advocacy group, paid or unpaid	__x__None	
11	Stock or stock options	_x___None	
12	Receipt of equipment, materials, drugs, medical writing, gifts or other services	_x___None	
13	Other financial or non-financial interests	__x__None	

Please place an "X" next to the following statement to indicate your agreement:

__X_ I certify that I have answered every question and have not altered the wording of any of the questions on this form.
